# Dynamics of Bioactive Compounds and Their Relationship with Antioxidant and Antimicrobial Activity in the Pulp, Peel, and Seeds of ‘Salak’ During Ripening

**DOI:** 10.3390/foods14203476

**Published:** 2025-10-12

**Authors:** Elena Coyago-Cruz, Gabriela Méndez, Johana Zúñiga-Miranda, Nubia Jami, Ramiro Acurio-Vásconez, Jorge Heredia-Moya

**Affiliations:** 1 Carrera de Ingeniería en Biotecnología, Universidad Politécnica Salesiana, Sede Quito, Campus El Girón, Av. 12 de Octubre N2422 y Wilson, Quito 170143, Ecuador; gmendez@ups.edu.ec (G.M.); racurio@ups.edu.ec (R.A.-V.); 2 Centro de Investigación Biomédica (CENBIO), Facultad de Ciencias de la Salud Eugenio Espejo, Universidad UTE, Quito 170527, Ecuador

**Keywords:** functional foods, *Salacca zalacca*, ABTS, DPPH, antibacterial activities, antifungal activities

## Abstract

Fruit is an important source of bioactive compounds, and making full use of them can contribute to the development of natural alternatives to microbial resistance. This study aimed to evaluate the dynamics of bioactive compounds and their relationship with antioxidant and antimicrobial activity in the pulp, peel, and seeds of *Salacca zalacca* at three stages of ripeness (M1, 30 days after flowering; M2, 90 days after flowering; and M3, 120 days after flowering). The physicochemical characteristics (weight, size, pH, soluble solids, titratable acidity, moisture, ash, and minerals) and the bioactive compounds (vitamin C, organic acids, carotenoids, chlorophylls, and phenolic compounds) were determined using liquid chromatography. Antioxidant activity was determined using the ABTS and DPPH methods, and antimicrobial activity was assessed against *Escherichia coli*, *Staphylococcus aureus*, *Pseudomonas aeruginosa*, *Streptococcus mutans*, *Candida albicans* and *Candida tropicalis.* The results showed that the pulp had the highest concentrations of malic acid (8018.6 mg/100 g DW in M1); the peel in M1 had the highest concentrations of chlorogenic (705.0 mg/100 g DW), caffeic (321.0 mg/100 g DW) and ferulic acids (173.5 mg/100 g DW); and the seeds had the highest levels of vitamin C (16.81 mg/100 g DW in M2). The pulp in M2 and M3 and the peel in M2 exhibited the highest antioxidant capacity (5.5 mmol ET/100 g DW by DPPH), as well as the most potent antimicrobial activity against *S. aureus* and *E. coli*. In conclusion, the peel, in addition to the edible pulp, represents a relevant source of bioactive compounds with potential applications in functional foods and natural products.

## 1. Introduction

Fruit is recognised for its multiple health benefits, which are attributable to its high content of bioactive compounds. These include dietary fibre, vitamins, minerals, and phytochemicals with antioxidant and anti-inflammatory properties. Consuming these compounds has been associated with a reduced risk of cardiovascular disease, obesity, type 2 diabetes, cancer, and inflammatory disorders [1,2]. In particular, Amazonian and tropical fruits, in particular, stand out for their high carotenoid content, which is associated with anti-inflammatory, anticoagulant, antiviral, immunomodulatory, and antioxidant activities. This contributes significantly to the prevention of non-communicable diseases [3].

Incorporating non-traditional fruits into the diet increases nutritional diversity and contributes to the preservation of cultural practices. However, many species are part of traditional diets but have been neglected in modern agriculture [4]. Several studies have shown that fruits and their by-products contain bioactive compounds, such as polyphenols, betalains, flavonoids, phenolic acids, carotenoids and terpenes, which have recognised antioxidant and antimicrobial properties [5]. Despite this, the peel and seeds of fruits, which account for 10–35% of their total weight, are often discarded as waste even though they often have greater biological activity than the pulp. Their valorisation therefore represents an opportunity for the development of functional foods, contributing to food security, environmental sustainability, and a circular bioeconomy [6,7].

In this context, the search for natural antimicrobials has gained importance given that antimicrobial resistance is currently a major global health crisis, exacerbated by the misuse of antibiotics in both humans and animals [8,9]. Bioactive compounds from fruits have thus emerged as potential therapeutic and preventive alternatives with applications in the food, pharmaceutical, and cosmetic industries. On the other hand, the ‘Salak’ (*Salacca zalacca* (Gaertn.) Voss), also known as the ‘snake fruit’, is a palm tree native to Southeast Asia that is mainly cultivated in Indonesia and Malaysia [10], but recently introduced into Ecuador, where it is gaining popularity in local markets [11]. The oval-shaped fruit, which averages 5–8 cm in length, grows in clusters at the base of the stem. Its reddish-brown, scaly peel characterises it; yellowish-white pulp with a sweet and sour taste; and three 2–3 cm seeds [12,13]. The pulp is rich in phenolic compounds, such as chlorogenic acid, epicatechin and isoquercetin, which provide antioxidant activity and help to reduce oxidative stress [14]. In certain parts of India, the tender seeds are also consumed [6]. Pulp is also consumed fresh and used in the production of probiotic beverages, which have been shown to inhibit the growth of *Escherichia coli*, further reinforcing its potential as a functional food [15].

Various ‘Salak’ varieties have been developed, each with different chemical and sensory characteristics that influence consumer acceptance and commercial value [16]. Recent studies have highlighted the fruit’s high content of flavonoids, polyphenols and other antioxidant compounds, including chlorogenic acid, which have anti-ageing and health-promoting properties [17]. However, research on ‘Salak’ remains scarce, particularly regarding the peel and seeds, and even more so concerning the variation in bioactive compounds during fruit ripening. While similar studies have been conducted on other tropical fruits, little is known about the dynamics of bioactive compounds in ‘Salak’ and their relationship with antioxidant and antimicrobial activity across different maturity stages. This knowledge is crucial not only for understanding the biochemical changes in the fruit but also for identifying potential applications of its by-products in the development of functional foods, nutraceuticals, and natural preservatives. Therefore, the objective of this study was to determine the dynamics of bioactive compounds and their relationship with antioxidant and antimicrobial activity in the pulp, peel, and seeds of ‘Salak’ at three different stages of ripeness.

## 2. Materials and Methods

### 2.1. Reagents and Standards

Chemical compounds studied in this article, such as acetone (CAS 67-64-1), phenolphthalein (CAS 77-09-8), sodium hydroxide (CAS 1310-73-2) and trichloromethane (CAS 67-66-3) were of analytical grade. At the same time, acetonitrile (CAS 75-05-8), ethanol (CAS 64-17-5), ethyl acetate (CAS 141-78-6) and methanol (CAS 67-56-1) were of HPLC-grade and purchased from Fisher Chemical (Fischer Scientific Inc., Madrid, Spain). ABTS (2,2-azino-bis-(3- ethylbenzothiazoline-6-sulphonic acid) (CAS 30931-67-0), benzene (CAS 71-43-2), chloroform (CAS 67-66-3), *DL*-homocysteine (CAS 454-29-5), DPPH (2,2-Diphenyl-1-picrylhydrazyl) (CAS 1898-66-4), ferric chloride (CAS 7705-08-0), formic acid (CAS 64-18-6), Mayer’s reagent (CAS 39775-75-2), metaphosphoric acid (CAS 37267-86-0), *n*-acetyl-*n,n,n*-trimethyl ammonium bromide (CAS 57-09-0), nitric acid (CAS 7697-37-2), potassium persulfate (CAS 7727-21-1), monobasic potassium phosphate (CAS 7778-77-0) and sulfuric acid (CAS 7664-93-9) were of analytical grade and purchased from Sigma (Merck, Darmstadt, Germany). Hydrochloric acid (CAS 7647-01-0) was of analytical grade and purchased from Labscan (RCI Labscan Group, Dublin, Ireland). Water was purified using a NANOpureDiamondTM system (Barnsted Inc., Dubuque, IO, USA). *L*-(+)-ascorbic acid 99.8% (CAS 50-81-7), citric acid 100.8% (CAS 77-92-9), malic acid 99.0% (CAS 97-67-6), *L*-(+)-tartaric acid 99.5% (CAS 87-69-4), caffeic acid 98.0% (CAS 331-39-5), chlorogenic acid 95.0% (CAS 327-97-9), chrysin 97.0% (CAS 480-40-0), *p*-coumaric acid 98.0% (CAS 501-98-4), *m*-coumaric acid 99.0% (CAS 588-30-7), *o*-coumaric acid 97.0% (CAS 614-60-8), ferulic acid 100.0% (CAS 1135-24-6), gallic acid 100.0% (CAS 149-91- 7), *p*-hydroxybenzoic acid (CAS 99-96-7), 3-hydroxybenzoic acid 99.0% (CAS 99-06-3), 2,5-dihydroxybenzoic acid 98.0% (CAS 490-79-9), kaempferol 97.0% (CAS 520-18-3), luteolin 98% (CAS 491-70-3), naringin 95.0% (CAS 10236-47-2), quercetin 95.0% (CAS 849061- 97-8), rutin 94.0% (CAS 153-18-4), shikimic acid 99.0% (CAS 138-59-0), syringic acid 95.0% (CAS 530-57-4), vanillic acid 97.0% (CAS 121-34-6), α-carotene >95.0% (CAS 7488-99-5), β-carotene 93.0% (CAS 7235-40-7), β-cryptoxanthin 97.0% (CAS 472-70-8), lutein (CAS 127-40-2), lycopene (CAS 502-65-8), zeaxanthin (CAS 144-68-3), and Trolox 98% (CAS 53188-07-1), were of standard grade and purchased.

For mineral analysis, standard solutions of iron (CAS 7439-89-6), sodium (CAS 7440-23-5), magnesium (CAS 7439-95-4), potassium (CAS 7440-09-7), and calcium (CAS 7440-70-2) at a concentration of 1000 µg/mL were obtained from Accustandard (AccuStandard, Inc., New Haven, CT, USA). In microbiological assays, culture media such as Mueller–Hinton agar (MHA), brain heart infusion (BHI), and Sabouraud dextrose agar (SDA) were sourced from BD Didcot (Fisher Scientific Inc., Madrid, Spain). Dextrose-yeast peptone broth (YPDB) was obtained from SRL (Sisco Research Laboratories Pvt. Ltd., Bombay, India), and streptomycin sulphate (CAS 3810-74-0) was acquired from Phytotech (PhytoTechnology Laboratories^®^, Lenexa, KS, USA). The bacterial strains used in this study included Pseudomonas aeruginosa (ATCC 9027), Streptococcus mutans (ATCC 25175), Staphylococcus aureus (ATCC 6538P), and Escherichia coli (ATCC 8739). The yeast strains were Candida albicans (ATCC 1031) and Candida tropicalis (ATCC 13803). All microbial strains were obtained from the American Type Culture Collection (ATCC) in Manassas, VA, USA. Ultrapure water used throughout the experiment was obtained using a NANOpure Diamond system (Barnstead Inc., Dubuque, IA, USA).

### 2.2. Physicochemical Analyses

**Figure 1:**
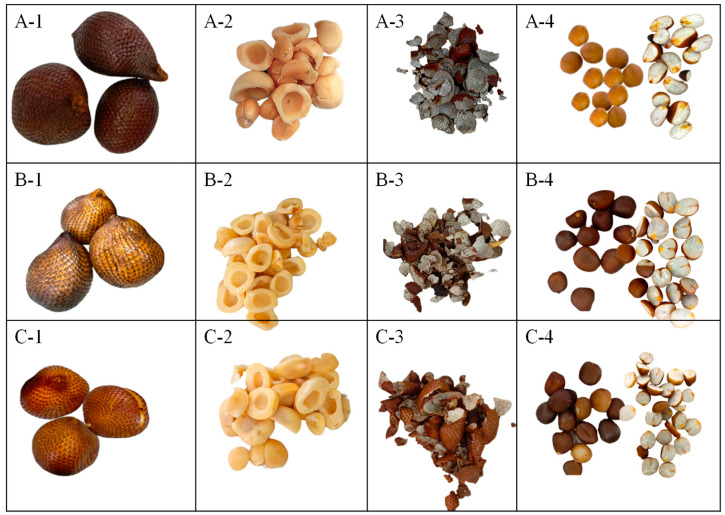
*Salacca zalacca* (‘Salak’) at different stages of maturity (4495, Herbarium QUPS-Ecuador). Note: (**A-1**), ‘Salak’ 30 days after flowering (M1); (**B-1**), ‘Salak’ 90 days after flowering (M2); (**C-1**), ‘Salak’ at 120 days after flowering (M3); (**A-2**–**C-2**), pulp to the respective stages of maturity; (**A-3**–**C-3**), peel to the respective stages of maturity; (**A-4**–**C-4**), seeds to the respective stages of maturity.

The ‘Salak’ sample was collected in the province of Pichincha in Ecuador (0°04’26.4” N 78°58’51.1” W). To facilitate botanical identification, the plant material was pressed and dried (Identification code: 4495, Herbario QUPS-Ecuador). Sixty fruits were randomly selected at various stages of phenological development (Figure 1), such as initial development (M1, 30 days after flowering); intermediate development (M2, 90 days); and commercial maturity (M3, 120 days) [11,18]. The sample was then divided into two portions. The first portion (fresh fruit) was used for physicochemical analyses. In contrast, the second portion was separated into pulp, peel, and seeds, which were freeze-dried using a Christ Alpha 1-4 LDplus (GmbH, Osterode am Harz, Germany) [19].

The weight of the thirty fresh fruits and seeds was determined using an ML204T/00 balance (Mettler Toledo, Greifensee, Switzerland). At the same time, the equatorial and longitudinal diameters were measured using a Titan 23175 calibrator from Titan (Kent, WA, USA). In pulp, peel and seeds, pH was measured using a SevenMulti™ S47 pH meter (Mettler Toledo, Greifensee, Switzerland); soluble solids were measured using a manual refractometer (Boeco, Hamburg, Germany) [20]; total titratable acidity was measured using acid–base titration; and moisture was measured using a Be20 oven (Memmert GmbH Co. KG, Schwabach, Germany) at 110 °C, while ash content was determined in a muffle furnace (Thermo Fisher Scientific, Waltham, MA, USA) at 550 °C [11,21].

#### 2.2.1. Mineral Determination

Mineral extraction was performed on 40 mg of freeze-dried sample from each fraction, separately. To each sample, 5 mL of 65% nitric acid was added. This mixture was left to stand in the digester for ten minutes, after which it was digested using a Speed-180 Wave Xpert Microwave (Berhof Products + Instruments GmbH, Eningen unter Achalm, Germany). The digested sample was diluted with deionised water to a final volume of 25 mL, after which it was stored in an amber glass container until analysis.

Mineral quantification was performed by atomic absorption spectrophotometry using a Varian SpectrAA-555 (Varian Inc., Palo Alto, CA, USA) with specific lamps for calcium (422.7 nm and 0.5 nm of slit), iron (372.0 nm and 0.20 of slit), potassium (404.4 nm, 0.5 nm if slit), magnesium (202.6 nm and 1.0 nm of slit), and sodium (589.6 nm and 0.5 nm slit). Calibration curves were prepared from 1000 ppm stock solutions of each standard. Each extraction was performed in triplicate, with duplicate readings taken for each sample. The results were expressed as milligrams of mineral per 100 g of freeze-dried sample (mg/100 g DW) [19].

#### 2.2.2. Phytochemical Screening

The extract was obtained by mixing 20 mg of the lyophilised sample with 1000 μL of deionised water. This mixture was homogenised using a VM-300 vortex mixer (Interbiolab Inc., Orlando, FL, USA) and stirred for 3 min in an FS60D ultrasonic bath with 130 W of power and a 40 kHz frequency (Fisher Scientific Inc., Waltham, MA, USA). The supernatant was recovered by centrifugation in an Eppendorf 5430 (Eppendorf AG, Hamburg, Germany) centrifuge at 14,000 rpm for five minutes at 4 °C. The solid residue was then subjected to a second extraction using 500 μL of water, following the procedure described above. Finally, the obtained supernatants were combined for the corresponding analysis.

The analysis covered alkaloids, acetogenins, anthraquinones, flavonoids, phenols, saponins, steroids, tannins, and terpenoids in accordance with the methodology outlined by León-Fernández et al. [22]. Terpenoids were analysed by reaction with chloroform and sulphuric acid, phenols, and tannins with a 10% ferric chloride solution, alkaloids with 2N hydrochloric acid and Mayer’s reagent, flavonoids with a 1 M solution of 10% ammonia and concentrated sulphuric acid, anthraquinones with benzene and 10% ammonia, saponins with the presence of foam in the mixture, and acetogenins with 3-5 dinitrobenzoic acid and potassium hydroxide. Each extraction was performed in triplicate, with duplicate readings taken for each sample. Qualitative results were expressed as ‘+’ when the reaction indicated the presence of the compound, and ‘-’ when it did not.

### 2.3. Analysis of Bioactive Compounds

#### 2.3.1. Vitamin C

The extract was obtained by mixing 20 mg of lyophilised powder with 1.2 mL of 3% metaphosphoric acid and 200 μL of 0.2% *DL*-homocysteine solution. The mixture was homogenised using a vortex mixer and then stirred for one minute in an ultrasonic bath. Then, 600 μL of deionised water was added, after which the supernatant was recovered by centrifugation at 14,000 rpm for 5 min at 4 °C. This supernatant was then filtered through a 0.45 µm PVDF filter and transferred to chromatography vials.

Quantification of vitamin C was performed using an RRLC 1200 chromatograph (Agilent Technologies, Santa Clara, CA, USA), equipped with a DAD-UV-Vis detector set to 244 nm, and a ZORBAX Eclipse XDB-C18 column (Agilent Scientific Instruments, Santa Clara, CA, USA) with a particle size of 1.8 μm and dimensions of 4.6 mm × 50 mm. The flow rate of the mobile phase was 1 mL/min. It was composed of a solution of 1.5% potassium monobasic phosphate and 1.8% *n*-acetyl-*n*,*n*,*n*-trimethylammonium bromide dissolved in HPLC-grade methanol (90:10 *v*/*v*). The calibration curve was prepared using standard solutions of *L*-(+)-ascorbic acid at a concentration of 1 mg/mL.

The chromatograms were analysed using the Lab ChemStation software (version 2.15.26) by comparing retention times and characteristic spectra. Each extraction was performed in triplicate and quantified in duplicate. The results were expressed as milligrams of vitamin C per 100 g of dry weight (mg/100 g DW) [23].

#### 2.3.2. Organic Acid Profile

The extract was obtained by mixing 20 mg of lyophilised powder with 1.5 mL of 0.02 N sulphuric acid containing 0.05% metaphosphoric acid and 0.02% *DL*-homocysteine solution. The mixture was homogenised using a vortex mixer and then stirred for 3 min in an ultrasonic bath. Then, 500 μL of deionised water was added, after which the supernatant was recovered by centrifugation at 14,000 rpm for 5 min at 4 °C. This supernatant was then filtered through a 0.45 µm PVDF filter and transferred to chromatography vials [24].

Quantification of vitamin C was performed using an RRLC 1200 chromatograph (Agilent Technologies, Santa Clara, CA, USA), equipped with a DAD-UV-Vis detector set to 210 nm, and a YMC-Triart C18 column (YMC Europe GmbH, Dinslaken, Germany) with a particle size of 3 μm and dimensions of 4.6 mm × 150 mm.

The flow rate of the mobile phase was 1 mL/min. It was composed of a solution of 0.027% sulphuric acid. The calibration curve was prepared separately using standard solutions of citric, malic, and tartaric acid at a concentration of 1 mg/mL.

The chromatograms were analysed using the Lab ChemStation software (version 2.15.26) by comparing retention times and characteristic spectra. Each extraction was performed in triplicate and quantified in duplicate. The results were expressed as milligrams of vitamin C per 100 g of dry weight (mg/100 g DW) [23].

#### 2.3.3. Carotenoid Profile

Carotenoid extraction was performed using 20 mg of freeze-dried powder mixed with 250 µL of methanol, 500 µL of dichloromethane and 250 µL of water. This mixture was then homogenised and stirred in an ultrasonic bath for two minutes. The coloured phase was then recovered by centrifugation at 14,000 rpm for three minutes at 4 °C. The solid residue was then subjected to successive extractions with 500 µL of trichloromethane until the solution became colourless. The coloured fractions were then combined and evaporated to dryness using a Büchi R-100 rotary evaporator (Fisher Scientific, Hampton, NH, USA) at 30 °C.

The dry extract was then reconstituted in 40 μL of HPLC-grade ethyl acetate before being transferred to an insert inside a chromatography vial. Analysis was performed using an RRLC 1200 system (Agilent Technologies, Santa Clara, CA, USA), equipped with a DAD-UV-Vis detector (250–500 nm) and a C18 Poroshell 120 column (2.7 μm, 5 cm × 4.6 mm) (Agilent Technologies). The mobile phase was pumped at a flow rate of 1 mL/min using a linear gradient: 0–5 min, 85% acetonitrile (A) + 15% methanol (B); 5–7 min, 60% A + 20% B + 20% ethyl acetate (C); 7–12 min, 85% A + 15% B.

A calibration curve was constructed using standard solutions of individual carotenoids at a concentration of 1 mg/mL, including β-carotene, β-cryptoxanthin, lutein, lycopene, zeaxanthin, astaxanthin, trans-β-apo-8′-carotenal, and α-carotene. The chromatograms were processed using the LabChemStation software (version 2.15.26), with 320 nm and 450 nm, by comparing retention times and characteristic spectra. Each extraction was performed in triplicate and quantified in duplicate. The results were expressed as milligrams of carotenoid per 100 g of dry weight (mg/100 g DW) [11].

#### 2.3.4. Chlorophylls and Their Derivatives

The extraction and analysis of chlorophylls and their derivatives were performed as outlined in Section 2.3.3. Standard solutions of chlorophyll a, chlorophyll b, pheophytin a and pheophytin b were prepared for quantification at a concentration of 1 mg/mL. Results were expressed as milligrams of carotenoids per 100 g of dry weight (mg/100 g DW).

#### 2.3.5. Phenol Profile

Phenolic compounds were extracted from 20 mg of freeze-dried powder by mixing it with 1000 µL of methanol that had been acidified with 0.1% HCl. The resulting suspension was homogenised and stirred in an ultrasonic bath for three minutes. The supernatant was recovered by centrifugation at 14,000 rpm for three minutes at 4 °C. The solid residue was then subjected to two successive extractions with 500 µL of acidified methanol. The resulting supernatants were combined and filtered through a 0.45 µm PVDF filter.

The filtered extract was transferred to chromatography vials and analysed using an RRLC 1200 system (Agilent Technologies, Santa Clara, CA, USA), which was equipped with a DAD-UV-Vis detector (200–500 nm) and a Zorbax Eclipse Plus C18 column (4.6 × 150 mm, 5 μm) (Agilent Technologies). The mobile phase was pumped at a flow rate of 1 mL/min using a linear gradient of 0.01% aqueous formic acid (solvent A) and acetonitrile (solvent B) according to the following programme: 0 min, 100% A; 5 min, 95% A + 5% B; 20 min, 50% A + 50% B; 30 min, column wash and re-equilibration.

The calibration curve was prepared using 1 mg/mL standard solutions of the following individual phenols: caffeic acid, chlorogenic acid, chrysin,*p*-,*m*- and *o*-coumaric acid, ferulic acid, gallic acid, *p*-hydroxybenzoic acid, 3-hydroxybenzoic acid, 2-methoxybenzoic acid, 3-methoxybenzoic acid, 2,5-dihydroxybenzoic acid, kaempferol, luteolin, naringin, quercetin, rutin, shikimic acid, syringic acid and vanillic acid.

The chromatograms were processed using LabChemStation software (version 2.15.26) by comparing retention times and characteristic spectra. Each extraction was performed in triplicate and quantified in duplicate. The results were expressed as milligrams of phenols per 100 g of dry weight (mg/100 g DW) [11].

### 2.4. Antioxidant Activity Analyses

The extract used to quantify antioxidant activity (ABTS and DPPH) was obtained by mixing 20 mg of lyophilised powder with 2 mL of methanol. The resulting suspension was homogenised and stirred in an ultrasonic bath for three minutes. The supernatant was then recovered by centrifugation at 14,000 rpm for three minutes at 4 °C, after which it was filtered through a 0.45 μm PVDF filter.

The ABTS radical was prepared by mixing 2.45 mM potassium persulfate with 7 mM ABTS. This mixture was left to stand in the dark for 16 h, after which it was diluted with methanol to an absorbance of 0.70 ± 0.02 at 734 nm. To quantify the sample, 10 μL of the sample or standard was added to each well of a 96-well microplate (VWR, Novachen, Pittsburgh, PA, USA), along with 200 μL of the ABTS radical solution. A calibration curve was constructed using standard trolox solutions diluted to a range of concentrations from 0.2 to 0.7 mM. Absorbance was measured at 734 nm using a microplate reader (Agilent Scientific Instruments, Santa Clara, CA, USA) in a spectrophotometer [25].

The DPPH radical was prepared by dissolving 10 mg of DPPH in 50 mL of HPLC-grade methanol. For analysis, 20 μL of the sample or standard was mixed with 280 μL of the radical solution in each well of a 96-well microplate. The plate was kept in the dark under agitation in a 4310-plate shaker (Fisher Scientific, Hampton, NH, USA) for 30 min. Absorbance was measured at 515 nm using a spectrophotometer with a microplate reader. A calibration curve was prepared using standard trolox solutions at 10 mM, diluted within the range of 0.4 to 4 mM.

Each extraction was performed in triplicate and quantified in duplicate. The results of both assays were expressed as millimoles of Trolox equivalent per 100 g of dry weight (mmol TE/100 g DW) [11,26].

### 2.5. Antimicrobial Activity Analyses

The antibacterial activity of ‘Salak’ extract was assessed against four bacterial strains, such as *Escherichia coli* ATCC 8739, *Pseudomonas aeruginosa* ATCC 9027, *Staphylococcus aureus* ATCC 6538P and *Streptococcus mutans* ATCC 25175 obtained from the American Type Culture Collection. Antimicrobial activity was evaluated using the microdilution method, as outlined in the Clinical and Laboratory Standards Institute (CLSI) guidelines, with specific modifications [27,28] and three repetitions. Bacterial strains were pre-cultured overnight in brain heart infusion (BHI) broth at 37 °C under rotary shaking to ensure exponential growth. Subsequently, cultures were adjusted to a standardised inoculum of 5 × 10^5^ CFU/mL. A stock solution of each extract was prepared by dissolving 300 mg of lyophilised material in 1 mL of distilled water. Serial two-fold dilutions of this stock were performed in water, and 180 µL of each dilution was mixed with 20 µL of bacterial suspension, yielding a final well volume of 200 µL in 96-well microplates. Plates were incubated at 37 °C for 24 h. After 2 h, 20 µL of 2,3,5-triphenyltetrazolium chloride (TTC) solution was added to each well and incubated for an additional 2 h at 37 °C. TTC is reduced to formazan by bacteria, producing a distinct red colour. Absence of colour indicated complete growth inhibition. The minimal inhibitory concentration was defined as the lowest concentration of extract (mg/mL) that completely prevents visible growth. All experiments were performed in triplicate.

The antifungal activity of ‘Salak’ extract was assessed against two *Candida* strains, such as *Candida albicans* ATCC 1031 and *Candida tropicalis* ATCC 13803, obtained from the American Type Culture Collection. Antifungal activity was evaluated using a standardised microdilution assay adapted from CLSI guidelines [27,28,29]. Lyophilised extracts were dissolved in sterile water to prepare a solution by dissolving 300 mg of dry extract in 1 mL of solvent. Fungal inocula were generated from 24 h cultures grown in Yeast Peptone Dextrose Broth (YPD) at 30 °C, then adjusted to a turbidity equivalent to 0.5 McFarland standard. Each well of the 96-well microplate received 180 µL of serially diluted extract combined with 20 µL of fungal suspension, resulting in a final volume of 200 µL per well. Plates were sealed and incubated at 30 °C for 72 h to allow for complete fungal growth or inhibition to occur.

To assess metabolic viability, 20 µL of 2,3,5-triphenyltetrazolium chloride was added to each well following incubation, followed by an additional 2 h incubation at 30 °C. TTC allows distinguishing between growth (red colouration) and inhibition (colourless wells). The MIC was defined as the lowest extract concentration that completely suppressed visible fungal growth. All experiments were performed in triplicate.

### 2.6. Statistical Analysis

Statistical analyses were performed using STATGRAPHICS Centurion XVII and RStudio (version 4.4.1). The results are expressed as the mean ± standard deviation (SD). ANOVA and Tukey’s multiple comparison test were performed at a significance level of *p* < 0.01. A principal component analysis (PCA) and a heatmap were performed to identify the variables most strongly associated with differentiation between maturity stages and fruit parts. These included the full range of evaluated variables, such as mineral content, vitamin C, carotenoids, phenols, organic acids, antioxidant capacity (as measured by ABTS and DPPH assays), and antimicrobial activity (expressed as the inhibition zone diameter in millimetres).

## 3. Results

### 3.1. Physico-Chemical Characteristics

The physicochemical characteristics of ‘Salak’ fruits were analysed, including the determination of weight and diameter (longitudinal and equatorial) at three ripeness stages, M1 (30 days after flowering), M2 (90 days) and M3 (120 days), as summarised in Table 1. The fruit’s weight increased progressively from 28.7 g in M1 to 67.1 g in M3, while the seeds showed no significant differences, averaging 4.4 g. The longitudinal diameter of the fruit increased from 44.1 mm in M1 to 55.9 mm in M3, while that of the seeds increased from 16.3 mm to 22.3 mm. Similarly, the equatorial diameter varied from 35.3 mm in M1 to 51.9 mm in M3, with a range of 15.3–21.2 mm for the seeds.

The results obtained for the weight, longitudinal diameter and equatorial diameter of the fruit increased progressively with the degree of ripeness. This reflects the expected physiological growth during fruit development. Other authors have suggested that the increase in weight is associated with changes in the water status and cellular structure of fruit tissues [30,31]. Furthermore, the observed weight and size values were higher than those reported in previous studies, which ranged from 2.5 to 10 cm for the fruit and from 2 to 3 cm for the seeds [6], as well as from 5 to 8 cm for the longitudinal range [12,13].

The pH value ranged from 3.3 in the pulp (M1 and M2) to 6.4 in the seeds (M1) (Table 2). In general, the seeds consistently recorded higher values than the pulp and peel at all three stages of ripeness, indicating lower acidity in this fraction.

Soluble solids ranged from 1.0 °Brix in the peel (M1) to 20.3 °Brix in the pulp (M3), with higher values observed in the pulp and rind than in the seeds. A significant increase in pulp was observed as ripening progressed; this is an effect associated with the reduction in other components, such as proteins and cell wall polymers, which are replaced by sugars, as other authors have noted [32,33].

Titratable acidity ranged from 0.1% in the peel (M2 and M3) to 0.9% in the pulp (M2). Unlike the peel and seeds, where no significant differences were detected between stages, the pulp exhibited the highest acidity values throughout the ripening process.

Moisture content fluctuated from 80.9% in the pulp (M1) to 56.7% in the peel (M3). These values are similar to those recorded in other Salak varieties (78.6–81.8%) [16], confirming the stability of this parameter.

Ash content ranged from 0.4% in the pulp (M1 and M2) to 3.1% in the peel (M3), being higher in the rind than in the pulp and seeds. This confirms its role as a mineral deposit. These values are consistent with those reported in previous studies (0.6 mg/100 g).

Overall, the pH range is consistent with that described for other Salak varieties (4.2–5.2). However, the soluble solids content was lower than that recorded for fruits grown in Indonesia (22.6–23.8 °Brix), but similar to that reported for fruits harvested in the Ecuadorian Amazon. These differences could be attributed to environmental and genetic factors. It has been documented that water stress influences the accumulation of sugars and organic acids by regulating genes and enzymes associated with carbohydrate and acid metabolism [34].

Regarding the mineral profile, calcium concentrations ranged from 92.2 mg/100 g DW in the pulp (M2) to 1199.7 mg/100 g DW in the peel (M1). Iron was undetectable in the pulp (M1 and M2) but reached 12.4 mg/100 g DW in the pulp (M3). Potassium ranged from 988.0 mg/100 g DW in the pulp (M2) to 1930.5 mg/100 g DW in the peel (M1). Magnesium ranged from 497.1 mg/100 g DW in the pulp (M2) to 1002.6 mg/100 g DW in the seeds (M1). Finally, sodium exhibited the highest concentrations, ranging from 2106.3 mg/100 g DW in the seeds to 16,384.5 mg/100 g DW in the peel (M3).

**Table 2:** Average values of the physicochemical characteristics of the ‘Salak’ pulp, peel, and seeds at different stages of maturity.

	Pulp			Peel			Seed					
	M1	M2	M3	M1	M2	M3	M1	M2	M3	A_M1_	A_M2_	A_M3_
pH	3.3 ± 0.1 ^b^	3.3 ± 0.1 ^b^	3.5 ± 0.0 ^a^	5.3 ± 0.0 ^a^	5.4 ± 0.1 ^a^	5.2 ± 0.0 ^a^	6.4 ± 0.1 ^a^	6.4 ± 0.1 ^a^	6.3 ± 0.0 ^a^	***	***	***
SS (°Brix)	15.3 ± 2.1 ^b^	19.0 ± 1.0 ^ab^	20.3 ± 1.5 ^a^	1.0 ± 0.0 ^b^	1.3 ± 0.6 ^b^	2.7 ± 0.6 ^a^	2.0 ± 0.0 ^a^	2.7 ± 0.6 ^a^	2.3 ± 0.6 ^a^	***	***	***
TA (%)	0.7 ± 0.1 ^ab^	0.9 ± 0.1 ^a^	0.7 ± 0.0 ^b^	0.2 ± 0.0 ^a^	0.1 ± 0.0 ^a^	0.1 ± 0.0 ^a^	0.2 ± 0.0 ^a^	0.2 ± 0.1 ^a^	0.2 ± 0.0 ^a^	***	***	***
Humidity (%)	80.9 ± 0.8 ^a^	79.5 ± 0.3 ^b^	80.0 ± 0.4 ^ab^	69.5 ± 4.9 ^a^	73.2 ± 2.5 ^a^	56.7 ± 4.0 ^b^	64.9 ± 0.2 ^a^	57.3 ± 0.1 ^b^	59.2 ± 1.7 ^b^	***	***	***
Ash (%)	0.4 ± 0.0 ^b^	0.4 ± 0.1 ^b^	0.6 ± 0.1 ^a^	1.9 ± 0.1 ^b^	1.3 ± 0.2 ^c^	3.1 ± 0.2 ^a^	0.9 ± 0.0 ^a^	0.9 ± 0.3 ^a^	1.2 ± 0.0 ^a^	***	*	***
Mineral profile (mg/100 g DW)												
Ca	124.8 ± 11.7 ^a^	92.2 ± 12.2 ^a^	111.5 ± 29.5 ^a^	1199.7 ± 154.9 ^a^	1105.5 ± 82.3 ^a^	1121.3 ± 59.9 ^a^	204.9 ± 8.8 ^a^	111.3 ± 1.7 ^b^	132.9 ± 21.3 ^b^	**	***	***
Fe	nd	nd	12.4 ± 0.4 ^a^	6.3 ± 0.1 ^b^	6.4 ± 0.6 ^b^	10.2 ± 0.1 ^a^	5.6 ± 0.8 ^a^	6.2 ± 0.4 ^a^	6.3 ± 0.7 ^a^	***	***	**
K	1197.2 ± 56.3 ^a^	988.0 ± 5.7 ^b^	1125.4 ± 34.0 ^ab^	1930.5 ± 263.8 ^a^	1462.9 ± 91.8 ^a^	1859.5 ± 120.2 ^a^	1073.9 ± 14.6 ^a^	1004.1 ± 62.4 ^a^	1066.5 ± 53.3 ^a^	*	**	**
Mg	606.5 ± 36.8 ^a^	497.1 ± 38.5 ^a^	499.2 ± 21.5 ^a^	782.4 ± 21.7 ^a^	900.1 ± 122.7 ^a^	864.0 ± 152.0 ^a^	1002.6 ± 34.8 ^a^	870.1 ± 75.1 ^a^	910.2 ± 148.6 ^a^	**	*	*
Na	9172.4 ± 69.8 ^a^	7009.9 ± 36.2 ^b^	6193.6 ± 45.2 ^b^	16,064.9 ± 194.3 ^a^	12,362.9 ± 97.9 ^a^	16,384.5 ± 6.3 ^a^	4164.1 ± 923.7 ^a^	2698.4 ± 167.8 ^a^	2106.3 ± 513.2 ^a^	**	**	***

Note: SS, soluble solids; TA, total titratable acidity; Ca, calcium; Fe, iron; K, potassium; Mg, magnesium; Na, sodium; M1, M2 and M3, ripeness stages at 30 days after flowering, 90 days, and 120 days, respectively. Different lowercase letters indicate significant differences between stages of ripeness within each part of the fruit under study. Asterisks indicate statistical differences between the various parts of the fruit at the same stage of ripeness: M1 (A_M1_), M2 (A_M2_), and M3 (A_M3_). nd, Undetectable; * indicates *p* < 0.1, ** indicates *p* < 0.01 and *** indicates *p* < 0.001.

The overall mineral profile was lower than in other reports where higher concentrations of potassium (11,339 mg/kg), calcium (220 mg/kg), magnesium (607 mg/kg), sodium (231 mg/kg) and iron (12.0 mg/kg) were observed in ripe pulp [12]. Nevertheless, this study confirmed that the peel contained higher concentrations of calcium, potassium, and sodium than the pulp and seeds, highlighting its potential as a rich source of minerals. The fact that this mineral accumulates in the shell is interesting, given that studies have shown accumulation in seeds [35,36].

Table 3 shows the results of the phytochemical analysis of the pulp, peel, and seed of the ‘Salak’ fruit at three stages of ripeness. The analysis involved the qualitative detection of steroids, terpenoids, phenols, tannins, alkaloids, flavonoids, anthraquinones, saponins, and acetogenins. The results revealed the presence of phenolic compounds and tannins in all parts of the ‘Salak’ fruit at every stage of ripeness.

Similarly, saponins were found in the pulp throughout the fruit’s development. These compounds are directly associated with antioxidant and antimicrobial properties, as well as potential protective effects against chronic diseases, as suggested by other authors [37]. Furthermore, their presence in the pulp could contribute to the reported antimicrobial activity, given that this group of compounds alters the permeability of microbial cell membranes and exhibits anti-inflammatory and antiparasitic properties [38]. The accumulation of saponins in the seeds may be responsible for their bitter taste, which could explain their use in traditional medicine, as suggested by other authors [39,40].

### 3.2. Analysis of Bioactive Compounds

Table 4 shows the average concentrations of bioactive compounds in the pulp, peel, and seeds of ‘Salak’ fruit at three stages of ripeness. The analysis included the vitamin C, organic acids (citric, malic, and tartaric), carotenoids, chlorophyll and its derivatives, and phenolic compounds. The antioxidant activity was also assessed using the ABTS and DPPH methods.

Vitamin C content ranged from 1.5 mg/100 g DW in the peel (M2) to 16.8 mg/100 g DW in the seeds (M2). The pulp and seeds had a higher concentration of vitamin C than the peel. However, the concentration of vitamin C found in this study was lower than that reported by other researchers, who indicated values of 400 mg/kg of ascorbic acid [12], as well as lower than that found in ‘Salak’ varieties grown in Indonesia, which ranged from 0.9 to 1.7 mg/g of pulp [16]. Previous studies did not detect the presence of vitamin C in ‘Salak’ grown in the Amazon [11].

Total organic acid content ranged from 155.7 mg/100 g DW in the seeds (M3) to 501.9 mg/100 g DW in the pulp (M1). Citric acid ranged from 73.7 mg/100 g DW in the peel (M3) to 322.8 mg/100 g DW in the seeds (M2), malic acid from 33.6 mg/100 g DW in the seeds (M2) to 7732.8 mg/100 g DW in the pulp (M1), and tartaric acid from 16.6 mg/100 g DW in the seeds (M2) to 501.9 mg/100 g DW in the pulp (M1). Thus, the pulp had the highest total organic acid content (the sum of citric, malic, and tartaric acids), surpassing the peel and seeds. The major compound among the organic acids in all three fractions (peel, pulp, and seeds) was malic acid. This is a characteristic shared with apples, which accumulate higher amounts of malic acid in their plant structures. However, it is worth noting that this characteristic may vary depending on the cultivar and environmental conditions [41]. Furthermore, previous studies of non-traditional fruits in Ecuador have revealed that this fruit contains high levels of malic acid [11].

In all cases, the values for carotenoids were below the detection limit. This study did not report carotenoid values, though other studies have found values of 5 mg/kg in ‘Salak’ pulp [12]. Other studies have reported the presence of lycopene (1130 μg/100 g) and β-carotene (29,997 μg/100 g) in fruit juice [42]. These discrepancies can be attributed to the wide genetic variability of ‘Salak’, which results in variations in the composition of bioactive compounds, as documented in various studies [11,12,16]. Furthermore, the results of this study were consistent with expectations: the pulp and seeds were opaque, which aligns with the literature indicating that specific carotenoids are associated with yellow, orange, and red hues [43].

Pheophytin b was only detected in the peel, with values ranging from 0.2 mg/100 g DW in M3 to 2.6 mg/100 g DW in M1.

Total phenolic compounds ranged from 26.6 mg/100 g DW in the seeds (M2) to 1201.3 mg/100 g DW in the peel (M1). Gallic acid, one of the individual compounds, ranged from 1.4 mg/100 g DW in the seeds (M2) to 11.6 mg/100 g DW in the pulp (M3). Syringic acid was absent from the pulp, but present in the peel and seeds, with values ranging from 4.5 mg/100 g DW in the seeds (M3) to 8.4 mg/100 g DW in the peel (M2). Chlorogenic acid ranged from 4.1 mg/100 g DW in the seeds (M2) to 705.0 mg/100 g DW in the peel (M1). Caffeic acid values ranged from 14.1 mg/100 g DW in the seeds (M2) to 321.0 mg/100 g DW in the peel (M1). Ferulic acid was undetectable in the seeds (M2), but present at levels of up to 173.5 mg/100 g DW in the peel (M1).

Phenolic profile, ‘Salak’ predominantly contains gallic, syringic, chlorogenic, caffeic and ferulic acids. ‘Salak’ pulp has been described as being rich in phenolic compounds, including chlorogenic acid, epicatechin and quercetin isomers [14,17], which is consistent with the results of this study. Similarly, other studies have reported pulp concentrations of up to 8.46 mg GAE/g [42] and 1300.9 mg/100 g DW [11]. However, it should be noted that the composition and structure of cell wall polyphenols can vary according to the fruit’s origin, which directly influences the concentration and characteristics of these metabolites [44]. A study that evaluated 51 non-traditional tropical species found that the concentration of phenolic compounds in ‘Salak’ was lower compared to other tropical fruits [11].

Antioxidant activity, as determined by the ABTS method, ranged from 0.6 mmol TE/100 g DW in the seeds (M2) to 4.1 mmol TE/100 g DW in the pulp (M2). Meanwhile, the DPPH method recorded values between 1.8 mmol TE/100 g DW in the peel (M1) and 5.5 mmol TE/100 g DW.

The antioxidant activity determined in this study was lower than that reported by other authors, who noted that ‘Salak’ pulp has a higher antioxidant capacity than other tropical fruits [11,12]. In vitro studies using the ABTS method have shown that the pulp can reach values of up to 260 mg ascorbic acid equivalents and 2.4 mg ascorbic acid per 100 g sample. The same study also found that the activity against the DPPH radical exceeded 250 mg equivalents of ascorbic acid per 100 g, showing a high correlation between the two tests and confirming the fruit’s antioxidant potency in relation to other local species [42].

Conversely, research on inedible fractions has revealed that ‘Salak’ peel can contain concentrations of up to 6.9 μg GAE/mg of extract [45], consistent with the findings of this study. This suggests that the peel is a significant source of phenolic compounds with antioxidant properties, as demonstrated by the ABTS and DPPH methods.

Overall, although the values obtained in this study were lower than those reported in the literature, the results suggest that the pulp and the peel of ‘Salak’ have a remarkable antioxidant profile. The magnitude of this profile may vary depending on varietal origin, growing conditions and the extraction and quantification methodology used, as suggested by other authors [23].

### 3.3. Antimicrobial Activity Analyses

Table 5 shows the minimal inhibitory concentration obtained when evaluating the antimicrobial activity of ethanolic extracts of the pulp, peel, and seeds, which were dried and redissolved in water, against bacteria (*Escherichia coli*, *Staphylococcus aureus*, *Pseudomonas aeruginosa* and *Streptococcus mutans*) and fungi (*Candida albicans* and *Candida tropicalis*) at both ends of the maturity scale (M1 and M3). The results showed that the pulp, peel, or seed extracts did not inhibit the growth of *C. albicans* or *C. tropicalis* at any stage of ripeness. However, *E. coli* and *S. aureus* in the pulp and peel were inhibited.

In the pulp, inhibition was observed against *E. coli*, *S. aureus*, *P. aeruginosa* and *S. mutans*, with MIC values ranging from 31.4 to 84.9 mg/mL depending on the stage of ripeness. The highest efficiency was recorded against *S. aureus* in M3 (31.4 mg/mL). These results suggest that the bioactive compounds present in the pulp, such as phenols and saponins, mainly inhibit Gram-positive bacteria. This is consistent with previous studies on the differential action of phenolic metabolites against microorganisms [37,38]. In this context, one study demonstrated the effectiveness of pulp against *E. coli* [15].

Antimicrobial activity was only detected in the peel against *E. coli* and *S. aureus*, with MIC values ranging from 42.1 to 84.3 mg/mL. The most notable inhibition was observed against S*. aureus* in M1 and M2 (42.1 mg/mL), which reinforces the role of this fraction as a reservoir of phenolic compounds associated with antimicrobial bioactivity. This is evident in Table 4, which shows that the peel has a higher concentration of phenolic compounds. However, no inhibition was observed against *P. aeruginosa*, *S. mutans*, *C. albicans* or *C. tropicalis*.

No inhibitory activity was recorded in any stage of seed maturity against either bacteria or fungi. This suggests that the metabolites present in this fraction either do not reach effective concentrations against the evaluated microorganisms or lack an antimicrobial effect. Thus, the low minimum inhibitory concentrations (MICs) of the extracts under study could be considered a viable alternative. Indeed, a review indicates that plant extracts with MICs of less than 8 mg/mL are considered active against microorganisms [46].

### 3.4. Statistical Analysis

**Figure 2:**
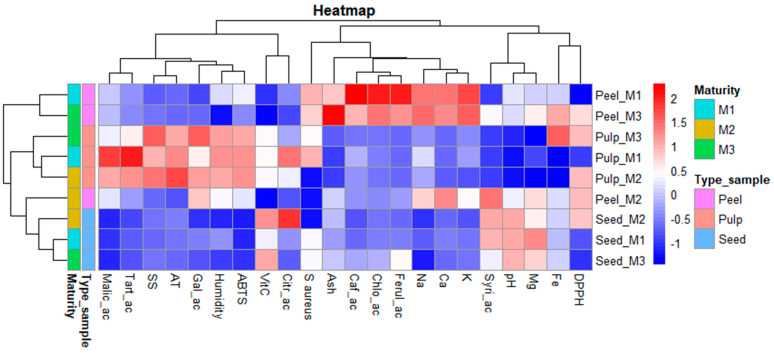
Heatmap of the pulp, peel, and seeds of the ‘Salak’ fruit. Note: SS, soluble solids; AT, total titratable acidity; Ca, calcium; Fe, iron; K, potassium; Mg, magnesium; Na, sodium; VitC, vitamin C; Citr_ac, citric acid; Malic_ac, malic acid; Tart_ac, tartaric acid; Gal_ac, gallic acid; Syri_ac, syringic acid; Chlo_ac, chlorogenic acid; Caf_ac, caffeic acid; Ferul_ac, ferulic acid; DPPH, antioxidant activity by DPPH; ABTS, antioxidant activity by ABTS; S.aureus, *Staphylococcus aureus;* M1, M2 and M3, ripeness stages at 30 days after flowering, 90 days, and 120 days, respectively.

Figure 2 shows the heatmap analysis for all the evaluated variables. This graph enables us to visualise patterns, correlations, and intensities between the variables under study. The heatmap illustrates the distribution and variability of various chemical compounds and physicochemical properties across different parts of the ‘Salak’ fruit, according to maturity stage. The analyses encompass three parts of the fruit as peel, pulp, and seed, also evaluated at three maturity levels (M1, M2, M3). The heatmap displays normalised values of malic acid, tartaric acid, soluble solids, titratable acidity, gallic acid, moisture content, ascorbic acid, sodium, calcium, potassium, syringic acid, pH, magnesium, iron, antioxidant activity, and antimicrobial activity against *S. aureus*. Colour intensity ranges from dark blue (low values) to bright red (high values), reflecting the relative magnitude of each variable. Dendrograms along both the horizontal and vertical axes indicate hierarchical clustering based on similarity among variables and samples, respectively.

The biochemical and functional characterisation of *S. zalazza* across its three fruit components, as peel, pulp, and seed, reveals dynamic, maturity-dependent modulation of bioactive compounds and associated biological activities. The maturity stages demonstrate significant implications for the fruit’s functional properties.

In the peel, M1 exhibits the highest concentrations of phenolic acids, including caffeic acid, chlorogenic acid, and ferulic acid. Compounds are widely recognised for their antioxidant capacity. At the M3maturity stage, ash content, a proxy of mineral density, peaks significantly, alongside sustained medium-high levels of chlorogenic acid and key minerals such as Na, K, Ca, and Fe. This suggests that while early peel is phenol-rich, mature peel evolves into a persistent phenolic content and a higher mineral content. In the seed, M1 shows only moderate levels of synergic acid, pH, and magnesium, with no notable biological activities reported. However, at M2 maturity stages, ascorbic acid and citric acid reach their highest concentrations. By M3, ascorbic acid declines to moderate levels, parallelled by reductions in Mg and pH, reflecting the lability of these compounds during late maturity.

The pulp, at the M1 maturity stage, is dominated by high levels of organic acids (malic, tartaric, citric), which contribute to its sour sensory profile and may partially underpin its moderate antioxidant and antimicrobial activities. By M2, titratable acidity peaks, phenolic content (gallic acid) and antioxidant capacity (both ABTS and DPPH) stabilise at medium levels. This is the stage where the strongest correlation between phenolic compounds and quantified antioxidant activity is observed, reinforcing the well-established link between phenolics and antioxidant activity.

**Figure 3:**
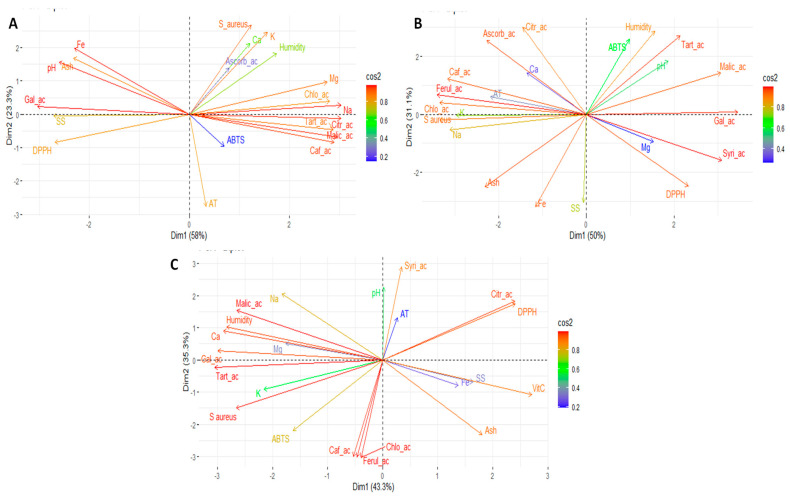
Principal component analysis (PCA) of the pulp, peel, and seeds of the ‘Salak’ fruit. Note: (**A**), PCA of the pulp; (**B**), PCA of the peel; (**C**), and PCA of the seeds; SS, soluble solids; AT, total titratable acidity; Ca, calcium; Fe, iron; K, potassium; Mg, magnesium; Na, sodium; VitC, vitamin C; Citr_ac, citric acid; Malic_ac, malic acid; Tart_ac, tartaric acid; Gal_ac, gallic acid; Syri_ac, syringic acid; Chlo_ac, chlorogenic acid; Caf_ac, caffeic acid; Ferul_ac, ferulic acid; DPPH, antioxidant activity by DPPH; ABTS, antioxidant activity by ABTS; S.aureus, *Staphylococcus aureus*.

Figure 3 presents the results of the principal component analysis (PCA) for all the evaluated variables. Figure 3A shows the corresponding PCA for the pulp, Figure 3B for the peel and Figure 3C for the seeds. The Principal Component Analysis (PCA) biplots provide a multivariate visualisation of the relationship among chemical constituents and biological activities measured across different samples of peel, seed, and pulp of ‘Salak’. In pulp PCA, the first two components explain 81.3% of the total variance. Dim 1 primarily separates samples based on their organic acid content, mineral composition, and antioxidant activity. Variables as malic acid, tartaric acid, citric acid, chlorogenic acid, caffeic acid and sodium are strongly positively correlated with Dim1.In contrast, titratable acidity is negatively associated with Dim1. Dim 2 captures variation related to phenolic content, pH, mineral composition, and antimicrobial activity. This suggests that higher phenolic content correlates with lower pH and reduced scavenging, which may reflect complex interactions between acidity and antioxidant mechanisms.

## 4. Conclusions

This study demonstrated that the dynamics of bioactive compounds in *Salacca zalacca* vary significantly depending on the fruit fraction (pulp, peel, and seeds) and the degree of ripeness. The pulp had a higher organic acid content, with malic acid being the predominant compound. In contrast, the peel was the main reservoir of phenolic compounds (chlorogenic acid, caffeic acid and ferulic acid), minerals (calcium, potassium, and sodium), and pheophytin B, the levels of which decreased with ripening. Although the seeds were less bioactive, they were rich in vitamin C. Antioxidant activity, as determined by the ABTS and DPPH methods, revealed that the pulp and peel exhibited the most significant capacity to neutralise radicals. In terms of antimicrobial activity, a moderate effect was observed against bacteria, primarily *Staphylococcus aureus* and *Escherichia coli*, in the pulp and peel. However, no inhibition was observed against fungi (*Candida albicans* and *Candida tropicalis*) in any of the three fractions studied. Overall, the results highlight the potential of not only the edible pulp but also the valorisation of by-products such as the peel, which is a concentrated source of phenols with antioxidant and antimicrobial properties.

## Figures and Tables

**Table 1:** Average values for the weight and size of the fresh ‘Salak’ fruit at different stages of maturity.

	Pulp			Seed					
	M1	M2	M3	M1	M2	M3	A_M1_	A_M2_	A_M3_
Weight (g)	28.7 ± 3.8 ^c^	46.2 ± 3.8 ^b^	67.1 ± 5.9 ^a^	4.1 ± 0.5 ^a^	4.3 ± 1.4 ^a^	4.9 ± 0.7 ^a^	***	***	***
LD (mm)	44.1 ± 3.4 ^b^	51.3 ± 4.6 ^ab^	55.9 ± 7.4 ^a^	16.3 ± 1.8 ^b^	21.5 ± 0.3 ^a^	22.3 ± 1.7 ^a^	***	***	***
ED (mm)	35.3 ± 8.9 ^b^	46.5 ± 1.4 ^a^	51.9 ± 2.7 ^a^	15.3 ± 1.7 ^b^	19.56 ± 0.3 ^a^	21.2 ± 2.7 ^a^	***	***	***

Note: LD, longitudinal diameter; ED, equatorial diameter. M1, M2 and M3, ripeness stages at 30 days after flowering, 90 days, and 120 days, respectively. Different lowercase letters indicate significant differences between stages of ripeness within each part of the fruit under study. Asterisks indicate statistical differences between the various parts of the fruit at the same stage of ripeness: M1 (A_M1_), M2 (A_M2_), and M3 (A_M3_). *** indicates *p* < 0.001.

**Table 3:** Phytochemical screening of the pulp, peel, and seeds of the ‘Salak’ fruit at different stages of maturity.

	Pulp			Peel			Seed		
	M1	M2	M3	M1	M2	M3	M1	M2	M3
Steroids	−	−	−	−	−	−	−	−	−
Terpenoids	−	−	−	−	−	−	−	−	−
Phenols	+	+	+	+	+	+	+	+	+
Tannins	+	+	+	+	+	+	+	+	+
Alkaloids	−	−	−	−	−	−	−	−	−
Flavonoids	−	−	−	−	−	−	−	−	−
Anthraquinones	−	−	−	−	−	−	−	−	−
Saponins	+	+	+	−	−	−	+	+	−
Acetogenins	−	−	−	−	−	−	−	−	−

Note: M1, M2 and M3, ripeness stages at 30 days after flowering, 90 days, and 120 days, respectively; +, positive to the reaction; −, negative to the reaction.

**Table 4:** Average values of the bioactive compounds and antioxidant activity of the ‘Salak’ pulp, peel, and seeds at different stages of maturity.

	Pulp			Peel			Seed					
	M1	M2	M3	M1	M2	M3	M1	M2	M3	A_M1_	A_M2_	A_M3_
Vitamin C (mg/100 g DW)	12.2 ± 0.6 ^a^	11.7 ± 1.12 ^a^	12.1 ± 0.0 ^a^	3.5 ± 0.2 ^a^	1.5 ± 0.1 ^b^	1.6 ± 0.1 ^b^	11.4 ± 0.6 ^b^	16.8 ± 0.9 ^a^	15.9 ± 0.6 ^a^	***	***	***
Organic acid (mg/100 g DW)												
Citric acid	283.9 ± 30.8 ^a^	187.0 ± 5.1 ^b^	137.8 ± 11.2 ^b^	118.7 ± 2.8 ^a^	83.7 ± 3.2 ^b^	73.7 ± 12.5 ^b^	121.7 ± 11.5 ^b^	322.8 ± 29.3 ^a^	87.0 ± 17.1 ^b^	**	**	*
Malic acid	7232.8 ± 840.6 ^a^	5459.0 ± 20.1 ^ab^	3650.3 ± 201.9 ^b^	2909.2 ± 21.9 ^b^	3387.3 ± 118.3 ^a^	2713.6 ± 132.3 ^b^	197.5 ± 8.6 ^a^	33.6 ± 1.1 ^b^	35.4 ± 2.6 ^b^	**	***	***
Tartaric acid	501.9 ± 105.6 ^a^	364.5 ± 10.3 ^a^	251.7 ± 13.1 ^a^	106.0 ± 0.5 ^a^	112.0 ± 4.4 ^a^	89.9 ± 0.6 ^b^	45.5 ± 1.9 ^a^	16.6 ± 3.8 ^c^	33.3 ± 2.0 ^b^	***	***	***
Total organic acid	8018.6 ^a^ ± 977.0 ^a^	6010.5 ± 216.7 ^ab^	4039.8 ± 226.3 ^b^	3133.9 ± 18.7 ^ab^	3583.9 ± 125.9 ^a^	2877.2 ± 145.4 ^b^	364.8 ^c^ ± 22.0 ^a^	373.1 ± 26.6 ^a^	155.7 ± 21.7 ^b^	**	***	***
Carotenoids (mg/100 g DW)		nd	nd	nd	nd	nd	nd	nd	nd			
Pheophytin b (mg/100 g DW)				2.6 ± 0.0 ^a^	0.7 ± 0.0 ^b^	0.2 ± 0.0 ^c^						
Phenolics profile (mg/100 g DW)												
Gallic acid	7.5 ± 0.5 ^b^	9.9 ± 0.5 ^a^	11.6 ± 0.4 ^a^	1.8 ± 0.1 ^b^	8.7 ± 0.6 ^a^	2.8 ± 0.0 ^b^	3.5 ± 0.0 ^a^	1.4 ± 0.1 ^c^	2.3 ± 0.2 ^b^	***	***	***
Syringic acid					8.4 ± 0.8 ^a^	4.8 ± 0.4 ^b^	6.8 ± 0.4 ^a^	7.1 ± 0.7 ^a^	4.5 ± 0.3 ^b^	***	ns	ns
Chlorogenic acid	12.2 ± 1.3 ^a^	9.0 ± 0.3 ^ab^	8.3 ± 0.4 ^b^	705.0 ± 18.9 ^a^	39.0 ± 3.5 ^b^	533.2 ± 39.4 ^a^	18.1 ± 0.3 ^b^	4.1 ± 0.1 ^b^	114.4 ± 12.2 ^a^	*	**	***
Caffeic acid	78.5 ± 4.9 ^a^	58.7 ± 1.5 ^b^	31.1 ± 2.3 ^c^	321.0 ± 69.8 ^a^	52.0 ± 3.3 ^b^	190.8 ± 8.7 ^ab^	23.8 ± 0.9 ^b^	14.1 ± 0.0 ^b^	71.2 ± 9.1 ^a^	***	***	***
Ferulic acid				173.5 ± 0.3 ^a^	26.3 ± 1.1 ^c^	123.8 ± 3.1 ^b^	11.4 ± 0.4 ^b^	nd	74.2 ± 4.7 ^a^	***	ns	**
Total phenolics	98.3 ± 6.8 ^a^	77.7 ± 1.3 ^b^	51.1 ± 1.5 ^c^	1201.3 ± 25.8 ^a^	134.32 ± 0.9 ^b^	855.5 ± 51.8 ^a^	63.6 ± 1.2 ^b^	26.6 ± 0.7 ^b^	266.7 ± 26.0 ^a^	**	***	***
Antioxidant activity (mmol ET/100 g DW)												
ABTS	4.0 ± 0.2 ^a^	4.1 ± 0.0 ^a^	3.8 ± 0.4 ^a^	2.7 ± 0.5 ^a^	2.7 ± 0.4 ^a^	1.7 ± 0.2 ^b^	0.8 ± 0.0 ^a^	0.6 ± 0.1 ^b^	0.9 ± 0.1 ^a^	***	***	***
DPPH	2.3 ± 0.1 ^b^	5.5 ± 0.7 ^a^	5.5 ± 0.4 ^a^	1.8 ± 0.3 ^a^	5.5 ± 0.5 ^a^	5.1 ± 0.9 ^a^	2.6 ± 0.5 ^a^	5.4 ± 0.8 ^a^	2.3 ± 0.1 ^b^	***	ns	***

Note: M1, M2 and M3, ripeness stages at 30 days after flowering, 90 days, and 120 days, respectively. Different lowercase letters indicate significant differences between stages of ripeness within each part of the fruit under study. Asterisks indicate statistical differences between the various parts of the fruit at the same stage of ripeness: M1 (A_M1_), M2 (A_M2_), and M3 (A_M3_). nd, undetectable; * indicates *p* < 0.1, ** indicates *p* < 0.01 and *** indicates *p* < 0.001.

**Table 5:** Minimal inhibitory concentration of the ‘Salak’ pulp, peel, and seeds at different stages of maturity.

		Minimal Inhibitory Concentration (mg/mL)					
		Bacterial Strain				Fungal Strain	
		*E. coli* ATCC 8739	*S. aureus* ATCC 6538P	*P. aeruginosa* ATCC 9027	*S. mutans* ATCC 25175	*C. albicans* ATCC 1031	*C. tropicalis* ATCC 13803
Pulp	M1	31.9	42.6	42.6	84.9	na	na
	M2	42.5	84.9	84.9	na	na	na
	M3	41.8	31.4	83.7	na	na	na
Peel	M1	84.3	42.1	na	na	na	na
	M2	84.2	42.1	na	na	na	na
	M3	83.3	83.3	na	na	na	na
Seed	M1	na	na	na	na	na	na
	M2	na	na	na	na	na	na
	M3	na	na	na	na	na	na

Note: na, non-active at the tested concentrations.

## Data Availability

The original contributions presented in the study are included in the article, further inquiries can be directed to the corresponding author.

## Abbreviations

The following abbreviations are used in this manuscript:

QUPS	Universidad Politécnica Salesiana Sede Quito
